# Cross-Cultural Variation in Political Leadership Styles

**DOI:** 10.5964/ejop.v13i4.1412

**Published:** 2017-11-30

**Authors:** Petia Paramova, Herbert Blumberg

**Affiliations:** a Department of Psychology, BPP University, London, United Kingdom; b Department of Psychology, Goldsmiths College, University of London, London, United Kingdom; Webster University Geneva, Geneva, Switzerland

**Keywords:** leadership, political leadership, transformational leadership, transactional leadership, passive/avoidant leadership, MLQ, cross-cultural

## Abstract

Guided by gaps in the literature with regard to the study of politicians the aim of the research is to explore cross-cultural differences in political leaders’ style. It compares the MLQ (Avolio & Bass, 2004) scores of elected political leaders (N = 140) in Bulgaria and the UK. The statistical exploration of the data relied on multivariate analyses of covariance. The findings of comparisons across the two groups reveal that compared to British political leaders, Bulgarian leaders were more likely to frequently use both transactional and passive/avoidant behaviours. The study tests Bass’s (1997) strong assertion about the universality of transformational leadership. It contributes to the leadership literature by providing directly measured data relating to the behaviours of political leaders. Such information on the characteristics of politicians could allow for more directional hypotheses in subsequent research, exploring the contextual influences within transformational leadership theory. The outcomes might also aid applied fields. Knowledge gained of culturally different leaders could be welcomed by multicultural political and economic unions, wherein understanding and allowances might aid communication.

Initial research in the area of behavioural leadership theory aimed to group behaviours under a common denominator (Likert 1961; Stogdill & Shartle, 1955). This led to the emergence of two major groups that characterised two distinct styles. An array of names has been used to label these groups, but there is currently common agreement that one of the styles contains task-oriented properties while the other contains person-oriented properties.

Research explored which of the two styles is superior (i.e. research by Bales, 1950; Lewin, Lippitt, & White, 1939; Likert, 1961; Stogdill & Shartle, 1955), with results proving largely fruitless (Gastil, 1994), as most scholars agree that a combination of the two provides the most robust explanation of leadership (Blake & Mouton, 1978; Sorrentino & Field, 1986). Burns (1978), however, refused to agree that a superior style is absent and looked to describe leaders who have emerged and ensured effectiveness in times of crisis. Such leaders produced positive results by instigating change and they were seen as transformational. Moreover, transformational leaders have been described as trustworthy, honest, motivational and encouraging (Den Hartog, House, Hanges, et al., 1999). Avolio and Bass (1988) and Bass and Avolio (1990) presented a more elaborate transformational leadership theory—the Full Range Model—wherein transformational leadership style is contrasted with transactional and passive/avoidant styles. Like Burns (1978), Avolio and Bass (1988) described transformational leaders as: (a) those who raise subordinates’ awareness of the importance of achieving commonly set goals; (b) those who encourage subordinates to disregard what interests them for the sake of the common goal; and (c) those who develop their subordinates’ needs to a higher level, wherein they feel autonomous yet affiliated with the group. Bass (1985) viewed the compared transactional leadership as descriptive of those leaders who recognise what subordinates want to get from their job, help subordinates get what they want (if their performance allows) and exchange rewards for effort. Additionally, in this model the ‘passive/avoidant’ style signified a ‘negative’ leadership style, which was used as a baseline anchor point (Avolio, 1999).

In general, transformational theory is considered to have had a large impact upon leadership as a scientific domain. According to Antonakis (2012), it helped leadership researchers by providing them with direction at a time when the field suffered much pessimism. It presented researchers with a set of behaviours that were considered superior for inducing effectiveness, compared to the set of behaviours measured in the so-called two-factor theories (i.e. task- and person-oriented behaviours). Moreover, the Full Range Model benefits from a specially designed test (the Multifactor Leadership Questionnaire), currently used to measure the frequency of behaviours associated with the three styles.

Bass (1997) insisted on the universal relevance of transformational leadership and studies have cited that it is the most desired (Den Hartog et al., 1999) and the most effective of styles (Barling, Weber, & Kelloway, 1996; Derue, Nahrgang, Wellman, & Humphrey, 2011; Geyer & Steyrer, 1998; Judge & Bono, 2000; Judge & Piccolo, 2004; Lowe, Kroeck, & Sivasubramaniam, 1996; Rowold & Heinitz, 2007). Despite support for the stability of transformational leadership across settings, some research suggests that in some respects it varies as a function of the environment. Even though contextual factors were not seen to affect the effectiveness and the desirability of the style they were seen to affect its pervasiveness. According to findings the occurrence of the style is contingent on organisational and cultural factors (Bruch & Walter, 2007; Jung, Bass, & Sosik, 1995; Kuchinke, 1999; Leong & Fischer, 2011; Ling, Simsek, Lubatkin, & Veiga, 2008; Lowe et al., 1996; Ozorovskaja, Voordijk, & Wilderom, 2007; Pawar & Eastman, 1997). Organisational structure, type, hierarchy level and size have been thought to affect the frequency of its use. Transformational leaders have been seen to emerge mainly in the upper levels (Bruch & Walter, 2007) of the hierarchy in organic (Bass, 1985) and public (Lowe et al., 1996) organisations of small sizes (Ling et al., 2008). Similarly, cultures experiencing crisis (Conger, 1999) are reported as providing better contexts for the emergence of transformational leaders. Additional culture-contingent variables, such as political structure (Alas, Tafel, & Tuulik, 2007; Ozorovskaja et al., 2007) and differential values (Jung et al., 1995; Leong & Fischer, 2011), have also played a role in determining pervasiveness. For instance, transitional democracies and collectivist/mastery-oriented cultures (Jung et al., 1995; Leong & Fischer, 2011), show more transformational behaviours in their leaders.

Nonetheless, despite the widespread exploration of influences on transformational leadership practices, gaps in the literature are present. There is a notable absence of studies of political leaders even though transformational leadership theory is rooted in Burns’s (1978) study of political leaders. The difficulty in approaching politicians could explain this trend. However, it does not fully justify the apparent near total absence of studies quantitatively exploring behaviours displayed by political leaders. Other gaps in the literature are also present. They concern the lack of studies considering all three styles within the Full Range Model. Trends relating to the pervasiveness of transactional leadership and the effects of context on it are not as clear, because, often, studies exploring transformational leadership have not contrasted its effects with those of transactional and passive/avoidant styles. The exploration of culture as a context variable also seems to have been limited and when investigated an interest in Western cultures is predominant. While helpful, only few studies explore transformational leadership (Den Hartog et al., 1999) in developing and transitional democracies, and their regional clusters lack full diversity. For instance, within their Eastern European post-communist cluster, Den Hartog et al. (1999) included only Central European cultures such as those of Poland, Hungary and the Czech Republic. These countries share some historical similarity with other post-communist cultures—like those of Bulgaria or Lithuania—which allows for some generalisations. However, one must generalise with caution because Brodbeck et al. (2000) noted differences between historically similar cultures, which did not always share close geographical proximity.

Due to the aforementioned gaps, the transformational theory literature could benefit from studies exploring the presence and dynamics of the Full Range Model styles (i.e. transformational, transactional and passive/avoidant; Bass & Avolio, 1995) in the area of political leadership. The inclusion of political leader MLQ (Multifactor Leadership Questionnaire) self-ratings from direct leader assessment, the assessment of the transformational, transactional and passive avoidant leadership frequency data and the examination of the effects of variables such as culture might allow for more complete knowledge of political leadership style. The research question therefore explores the variance in the pervasiveness of the Full Range leadership style behaviours across cultures. A comparison between UK (developed Western democracy) and Bulgaria (developing post-communist South-Eastern European democracy) is made. In general, the comparison of Bulgaria to an established Western democracy could inform the hypothesis formation of future research by underlining the difference in leadership variables in Western and South-Eastern Europe. The comparison could also reveal much about the differences and similarities between cultures with varying historical, political and cultural profiles. Any knowledge of the differences and similarities between Bulgarian and British leaders could possibly be welcomed by structures such as the European Union, where understanding and allowances in communication could aid collaborative work in areas such as immigration. Furthermore, the findings could inform transformational theory by discussing variables which might have influence upon it and by noting the extent to which the Full Range model styles vary across cultural contexts.

The results of past research (Jung et al., 1995) describe leaders in collectivist cultures and cultures in critical conditions (Conger, 1999)—such as Bulgaria—as more transformational in nature. Ozorovskaja et al. (2007) asserted that post-communist leaders—such as those in Bulgaria—practice more transactional leadership behaviours, which as cited, is tied to high power distance (inequality) and authoritarianism. Based on such findings the expected outcome is for *Bulgarian political leaders to rate themselves as displaying higher levels of both transformational and transactional leadership behaviours, compared to British leaders. In relation to passive/avoidant leadership we could expect cross-cultural equality, as both cultures are presumed equally likely to avoid any association with salient indicators of ‘bad leadership’, however due to the lack of findings it could be wise to form a question--as opposed to hypothesis—asking whether there are cross-cultural differences in passive/avoidant leadership style behaviours displayed by political leaders.*

## Method

### Procedure

The data collection was carried out in a single phase. Invitations to participate were sent via e-mails to members of parliament and local councils in London and Sofia, the respective capitals of the United Kingdom and Bulgaria. The letters described the purpose of the study (i.e. a study looking to explore cross-cultural leadership practices)—without revealing the hypotheses—and provided recipients with the researcher’s contact details. In addition, they assured all potential participants’ personal data would be kept confidential. A total of 1,756 invitations were sent to political leaders. The response rate at this stage (suggesting interest in participation) was 9.1%. Those who expressed interest were then sent hard copies of the questionnaires, which were accompanied by an informed consent form. Following the receipt of the questionnaires the response rate fell to 8%. This is considerably low, but similar to the response rates achieved by other researchers using similar samples (e.g. the response rate in Caprara, Barbaranelli, Consiglio, Picconi, & Zimbardo’s [2003] study stood conservatively at 10%). The response rate is however likely be higher if one had accounted for e-mails not received or eliminated by filtering, as well as lost and forgotten questionnaires. Nevertheless, substantial systematic cross-cultural data for existing major political leaders are, as noted, fairly rare in the literature, because such data are difficult to obtain.

There was a considerable delay in response due to political leaders’ work commitments. Some declined immediately, expressing concerns over confidentiality and the way in which the data would be used.

The participants responded to a number of questionnaires (three closed and standardised questionnaires and one open-ended questionnaire) as part of a large investigation. All political leaders completed the self-form of the MLQ (Multifactor Leadership Questionnaire), which provided data on the style they utilise in addition to demographic information including nationality, gender, age, and political ideology, level of education, political post and voting behaviour.

### Participants

The sample was made up of elected political leaders (*n* = 140) currently holding office positions (i.e. as members of parliament or as local councillors). Within the sample, 63 of the leaders were from Bulgaria and 77 were from the United Kingdom. Table 1 represents demographic and political characteristics of this sample, arranged by nationality, age, gender, education, political involvement and political ideology.

**Table 1:** Political Leader Demographic Characteristics

Characteristic	Bulgarian (*n* = 63)		British (*n* = 77)		Total (*N* = 140)	
	*n*	%	*n*	%	*n*	%
Age						
18–37	11	17.5	14	18.2	25	17.9
38–57	41	65.0	32	41.5	73	52.1
58+	11	17.5	31	40.3	42	30.0
Gender						
Male	40	63.5	55	71.4	95	67.9
Female	23	36.5	22	28.6	45	32.1
Education						
Secondary	0	0.0	2	2.6	2	1.4
Further	14	22.2	8	10.4	22	15.7
Higher	49	77.8	54	70.1	103	73.6
Unknown	0	0.0	13	16.9	13	9.3
Level of political activity						
Local government	38	60.3	59	76.6	97	69.3
MP	25	39.7	18	23.4	43	30.7
Political ideology						
Left	14	22.2	26	33.8	40	28.6
Right	21	33.3	27	35.0	48	34.3
Centre	5	8.0	17	22.1	22	17.7
Unknown	23	36.5	7	9.1	30	21.4

*Note.* Percentages refer to the respective column.

### Research Tools

#### The MLQ (Multifactor Leadership Questionnaire – Overview

As suggested previously, many conceptualisations of leadership styles have been uncovered. Early models were particularly interested in testing task- and person-oriented leadership styles (e.g. Stogdill & Shartle’s Ohio State studies and Likert’s Michigan State studies, both in the 1950s), but, as neither style proved superior, interest shifted to a style that initiated and facilitated transformation in a positive manner.

This led to the development of the Full Range Leadership Model (Avolio & Bass, 1988; Bass & Avolio, 1990). The Full Range Model describes a broader range of leadership styles than the paradigms of initiation (task-oriented) and consideration (person-oriented) and uses the ‘Multifactor Leadership Questionnaire’ (MLQ; Avolio & Bass, 2004; Bass, 1985; Bass & Avolio, 1995, 2000) to measure the transformational, transactional and passive/avoidant styles.

The questionnaire is the most commonly employed measure of the three styles. It currently exists in four forms, under the names of ‘MLQ-5X-Short’ (leader and rater form) and ‘MLQ-5X-Long’ (leader and rater form). The former includes 45 items, while the latter includes 65 items. The 12 components of the 5X-Short version, used in this research, measure key leadership and effectiveness behaviours empirically linked to individual and organisational success. Five of the components describe transformational leadership (idealized attributes, idealized behaviours, inspirational motivation, intellectual stimulation and individualized consideration), two describe transactional leadership (management by exception–active and contingent reward) and two measure passive/avoidant leadership (management by exception–passive and laissez-faire). The remainder (three components, in total) deal with outcomes of leadership such as extra effort, effectiveness and satisfaction with leadership.

When presented with the leader form, leaders completing the questionnaire are usually asked to describe their style by judging how frequently each of the 45 statements fit them. Items are rated on a 5-point scale, anchored with 0 (not at all) and 4 (frequently, if not always).

The MLQ is considered a valid measure of the three leadership styles (i.e. transformational, transactional and passive/avoidant), with alpha coefficients for each component ranging from .74 to .94. Such coefficients suggest good internal consistency, and the items appear to measure the components which they claim to measure (Bass & Avolio, 1995). Even though Bass and Avolio (1995) presented the components of each style as independent (even *within* each style), high positive correlations (i.e. correlations above .8) have been noted, especially between transformational style components (Bass & Avolio, 1995). Such high correlations might not be welcomed by the questionnaire designers, as they suggest a level of component redundancy. However, some studies have used component integration, which usually corrects for this issue (Hetland & Sandal, 2003; Ross & Offermann, 1997). High significant positive correlations have also been found between the five components of the transformational style and the contingent reward component of the transactional style. Whilst not considered strength, Avolio and Bass (2004) suggested that the high association between the components is caused by both components representing active and positive forms of leadership. In addition, these correlations could be due to the claimed ability of leaders to be both transformational and transactional (Avolio & Bass, 2004).

#### Rationale for Choosing the MLQ

While there are many leadership style questionnaires (e.g. the Leadership Behaviour Description Questionnaire [LBDQ; Hemphill & Coons, 1957]; the Transformational Leadership Behaviour Inventory [TLI; Podsakoff, MacKenzie, Moorman, & Fetter, 1990]; and the Transformational Leadership Questionnaire [TLQ; Alimo-Metcalfe & Alban-Metcalfe, 2001]), the MLQ is, according to Avolio and Bass (2004), superior and widely used for targeting the measurements of transformational leadership and was therefore selected for this study.

In addition, while many of the other leadership questionnaires originated in areas other than politics, the MLQ relates to the work of Burns (1978), who discussed the idea of transformational leadership, following observations of political leaders. This made the MLQ appropriate for studying the present political leader sample.

Moreover, the structure of the MLQ has been cross-culturally replicated (Bass & Avolio, 1995). Widespread usage has resulted in many tested translations of the questionnaire, including a Bulgarian translation. In general, standardised measures of any kind are rarely employed in countries such as Bulgaria, posing problems for cross-cultural researchers keen to validly explore the constructs these questionnaires measure. The availability of a tested Bulgarian translation of the MLQ made it a suitable option from within the large array of available leadership behaviour tests.

The test has also been warranted with good internal consistency making issues of exceedingly high transformational component correlations easier to discount. The availability of a short form was also welcomed, given the difficulties associated with testing political leaders and the difficulty associated with introducing multiple measures.

## Analyses and Results

In order to explore the likely variations, political leader frequency scores on the MLQ scales (i.e. five transformational leadership scales, two transactional leadership scales and two passive/avoidant leadership scales) within the two cultural samples were compared. The preferred test for these comparisons was MANCOVA allowing for the exploration of multiple dependent variables and therefore reducing the likelihood of type I error. MANCOVA also addresses the issue of covariate effects, often leading to an increase in within-group error. Aspects such as gender (Eagly, Johannesen-Schmidt, & van Engen, 2003; Eagly & Johnson, 1990) and age (Kobacoff & Stoffey, 2001; Oshagbemi, 2008) have been previously shown to affect the frequency of leadership behaviours displayed by leaders; since these aspects were measured here, controlling for their effects helped when inferences about group variation were drawn. Further variables, such as ‘political ideology’ could also bring on confounding effects. Unfortunately, the large presence of missing data (see Table 1), associated with this variable prevented us from including it in the analysis. In general the inclusion of unreliable variables in MANCOVA is discouraged, as degrees of freedom and therefore power is lost with each addition of a variable. Political ideology should nevertheless be considered in future work.

While exploring assumptions for carrying out MANCOVA, some of the requested analyses showed sizeable correlations between scales belonging to each of the measured styles. The finding posed problems similar to those experienced by others (e.g., Hetland & Sandal, 2003). In order to solve the possible presence of redundant dependent variables, variable integration was undertaken. Combining the frequency scores of the five transformational scales into a single rating variable called the ‘transformational leadership frequency score’ and combining the scores of the transactional and passive/avoidant scales into two more single score variables (i.e. the ‘transactional leadership frequency score’ and the ‘passive/avoidant leadership frequency score’) was something other scholars in the area had done (Hetland & Sandal, 2003). The combination of variables reduced the original nine leadership style frequency variables into three new dependent variables: transformational, transactional and passive/avoidant leadership frequency scores. Before this combination, exploratory factor analysis with Varimax orthogonal rotation was carried out to investigate (independently of prior expectations) the higher order factor structure of the MLQ, in order to confirm the five transformational, the two transactional and the two passive/avoidant scales loaded onto three separate factors. The need for this was further prompted by evidence (Ardichvili & Kuchinke, 2002; Avolio & Bass, 2004; Geyer & Steyrer, 1998; Hetland & Sandal, 2003) suggesting that the transactional leadership scale contingent reward is highly correlated with all transformational leadership scales. Exploring the factor loadings was therefore necessary before variable integration took place.

The emerged factor structure following the exploratory factor analysis is illustrated in Table 2.

**Table 2:** Rotated Factor Matrix for the Factor Analysis of the MLQ Leadership Behaviour Scales

MLQ Scale	Factor 1	Factor 2	Factor 3
Idealised attributes^a^	**.724**	-.023	.189
Idealised behaviours^a^	**.779**	.068	.117
Inspirational motivation^a^	**.814**	-.104	-.154
Intellectual stimulation^a^	**.686**	-.013	.086
Individualised consideration^a^	**.684**	-.158	.064
Contingent reward^a^	**.674**	.006	.**378**
Management by exception–active^b^	.149	.102	**.949**
Management by exception–passive^c^	.021	**.894**	.093
Laissez-faire^c^	-.129	**.890**	.017
**% Variance** (Total: 66.46)	37.54	19.10	9.82

*Note.* Loadings in bold in each column signify that their respective scale loads highly on one of the three factors: Factor 1, Transformational Leadership style; Factor 2, Passive/avoidant leadership style; Factor 3, Transactional Leadership Style.

^a^transformational leadership style. ^b^transactional leadership style. ^c^passive/avoidant leadership style.

As presented in Table 2, the results note that, while the five transformational scales and two passive/avoidant scales loaded onto their respective factors, the transactional leadership scale contingent reward loaded quite highly onto both the transformational leadership factor and the factor containing the remaining transactional leadership scale management by exception–active.

It can, therefore, be argued that contingent reward should be included in the combined transformational leadership frequency score variable, rather than in the combined transactional leadership variable, where it is thought to belong. However, past research (Hetland & Sandal, 2003) has noted otherwise and, despite its high loading onto the transformational leadership factor, contingent reward was still included in the transactional factor during variable combination. This common practice has been supported by the test publishers and by other researchers (Avolio & Bass, 2004; Hetland & Sandal, 2003), who insist contingent reward is applicable to both types of leadership and note its high loading onto two of the three leadership behaviour factors/styles. Based on this, contingent reward was included in the transactional leadership frequency score variable.

Moreover, in terms of the remaining assumptions the data were suitable for a MANCOVA. Outliers were absent and all three dependent variables appeared as normally distributed for each level of the IVs (i.e. all skewness and kurtosis values were lower than the more stringent cut-off value of +/-1) (see Table 3 and 4 for descriptive statistics and skewness and kurtosis values). Missing values associated with the dependent variables (i.e. the three leadership styles) were not present and as described in Table 1 such were only evident with regard to the demographic variables. Heterogeneity was also absent, with all Levene’s tests suggesting insignificance (see Table 5).

**Table 3:** Means and Standard Deviations for the Three MLQ Leadership Style Frequency Scores for Bulgarian (N = 63) and British (N = 77) Political Leaders

Dependent variable	Bulgarian political leaders		British political leaders	
	*M*	*SD*	*M*	*SD*
Transformational leadership	3.05	0.42	2.92	0.53
Transactional leadership	3.13	0.49	2.47	0.57
Passive/avoidant leadership	1.28	0.58	0.87	0.60

**Table 4:** Ranges and Skewness and Kurtosis Values for the Three MLQ Leadership Style Frequency Scores for Bulgarian (N = 63) and British (N = 77) Political Leaders

Dependent variable	Bulgarian Political Leaders				British Political Leaders			
	Skewness Value	Kurtosis Value	Range		Skewness Value	Kurtosis Value	Range	
			Min	Max			Min	Max
Transformational leadership	-0.39	0.10	2.00	3.95	-0.41	-0.50	1.65	3.85
Transactional leadership	-0.18	-0.59	2.00	4.00	-0.03	0.25	0.75	3.75
Passive/avoidant leadership	0.31	0.37	0.00	3.00	0.93	0.86	0.00	2.75

**Table 5:** Levene’s Test Statistics for the Three MLQ Leadership Style Frequency Scores

Dependent variable	*F*	*p*
Transformational leadership	3.41	.07
Transactional leadership	1.11	.30
Passive/avoidant leadership	0.14	.71

The results of the multivariate analysis of covariance revealed a significant main effect of culture, *F*(3, 134) = 26.94, *p* < .001.

As hypothesised the follow up univariate analyses confirmed that Bulgarian and British political leaders varied with regard to their transactional, *F*(1, 136) = 59.82, *p* < .001, and unexpectedly with regard to their passive/avoidant style scores, *F*(1, 136) = 15.18, *p* < .001. A Bonferroni correction to the alpha level was employed to prevent the type I error usually associated with multiple follow-up analyses. On both occasions, Bulgarians appear to have scored higher (*M* = 3.13, *SD* = 0.49; *M* = 1.28, *SD* = 0.58, respectively), compared to UK nationals (*M* = 2.47, *SD* = 0.57; *M* = 0.87, *SD* = 0.6, respectively) (see Table 3 for all means and standard deviations). No significant differences were found with regard to the transformational style where Bulgarian and UK political leaders appeared to score similarly.

In addition, the effect sizes can be considered too. Where significance was reported, effect sizes (signified by eta squared [η^2^]) were medium to large (Cohen, 1988) (see Table 6 for η^2^).

**Table 6:** Effect Sizes Associated With Significant Main Effects After Analysis of Covariance

Political leader self-reported leadership style comparison	Eta squared (η^2^)	% of variance accounted for
Transactional style—significant main effect of culture	0.296***	29.6%
Passive/avoidant style—significant main effect of culture	0.099**	9.9%

*small effect size (η^2^ > 0.01), **medium effect size (η^2^ > 0.06), ***large effect size (η^2^ > 0.14).

## Discussion

The results of the analysis of culture differences in leadership styles partially supported the hypotheses. They suggest that, compared to British leaders, Bulgarian leaders scored significantly higher only in terms of transactional and passive/avoidant leadership style behaviours. While the transactional style differences were predicted, the lack of transformational style differences was unexpected. Moreover, the higher instances of passive/avoidant behaviours in Bulgarian leaders is an interesting and noteworthy finding, which tentatively answers the question posed in the introduction.

In order to explain the transactional leadership difference, one could explore the cultural value differences across the cultures studied. Bulgaria, for example, has been found to score highly in terms of power distance (Minkov, 2011), promoting authoritarianism, steep hierarchy, obedience towards those at the top, centralised power and reduced concern for employees in work settings. Some of these aforementioned concepts—such as authoritarianism—are associated with task-oriented behaviours--also related to, and present in, transactional leaders. In this way, one can associate power distance with the elevated levels of transactional behaviours in Bulgaria found in this research.

The provision of explanations for the passive/avoidant style variation is more challenging. It was expected that behaviours detrimental to the completion of a task are equally and universally absent in the leadership arena. Nevertheless, while passive/avoidant behaviours were still negatively associated with leadership in Bulgaria, they were certainly more frequently enacted by Bulgarian, than by British, leaders. No studies have looked at this in Bulgaria, but studies exploring countries with similar historical challenges—like Russia—have generated results similar to those obtained here. Ardichvili and Kuchinke’s (2002) findings presented low scores on the passive/avoidant leadership style scales laissez-faire and management by exception in all of the tested cultures. However, the scores were substantially higher for leaders from Russia, Georgia, Kazakhstan and Kyrgyzstan, compared to leaders from Germany and the USA. Similarly, Puffer (1996)—who studied the leadership styles of Russian leaders—suggested that these leaders display a collectivist attitude characterised by diffusion of responsibility (Mazar & Aggarwal, 2011), and a tendency to delegate decision making due to the wish to avoid the responsibility associated with unforeseen circumstances. A similarity between collectivism-based diffusion of responsibility and passive/avoidant leadership is evident, as both concepts are associated with decision making avoidance, delayed response and failure to interfere when needed. It is therefore likely that a high score on one (e.g. collectivism) could lead to a high score on the other (i.e. passive/avoidant leadership). Bulgaria’s more collectivist nature (Minkov, 2011) could explain its higher experience of inadequate leadership practices. Moreover, after looking at denial and avoidance of threat-related information, Metselaar (2012) described governments with authoritarian experiences—such as governments in Bulgaria—as more likely to tolerate denial and avoidance, compared to stable democratic systems.

Interestingly, even though a difference in the enactment of transformational leadership behaviours in Bulgaria and the UK was expected, such a difference was absent. The findings oppose those who characterise cultures in more critical environments (Conger, 1999), cultures with collectivist values (Jung et al., 1995) and cultures with authoritarian experiences (Eisenstadt, 1968), as displaying higher instances of transformational leadership. In general, charisma—a crucial aspect of transformational leadership—is often treated as an anti-democratic force and its heightened presence in cultures with history of totalitarian ruling was therefore expected. One possible answer to why leaders in Bulgaria and the UK scored similarly in terms of the practiced transformational leadership behaviours and therefore opposed what was hypothesised can be derived from Weber’s (1978) work. According to Weber, charisma can be equally developed in both dictatorial/autocratic and democratic situations. Weber (1978) described charismatic appeal as highly interactive with bureaucratic administration, but also as a concept which had democratic ramifications. Similarly, according to Gerth and Mills (1946), charisma can be ‘the vehicle of man’s freedom in history’; these researchers also suggested that, depending on its routinisation, charisma can exist equally in both democratic and undemocratic settings. Moreover, Bass (1997) agreed that transformational leadership can be both autocratic and directive, and democratic and participative. As noted earlier, he also advocated for the universality of transformational behaviours. Propositions as such could explain why transformational leadership appeared equally in Bulgaria and the UK.

### Limitations

The work is however not short of challenges. A theoretical area that could raise criticisms relates to the labels applied here. Many would note that calling elected officials ‘political leaders’ is actually unreasonable. The achievement of rising to public office might not be sufficient for convincing others of one’s leadership abilities. Thus, the use of electoral success as a proxy for leadership might be considered conceptually flawed, because, according to Burns (1978), we need to distinguish between leaders and power holders. However, there is no formal criterion for distinguishing ‘real’ from ‘non-real’ leaders, and the discussion surrounding the relationship between leader ‘position’ and ‘behaviour’ is exceedingly complex. Blondel (1987) noted that, often, one can have the position and the power, but not the behaviour that signifies leadership; however, while a distinction between the two must be made, he also agreed they affect each other. Leadership, according to Blondel (1987), is the product of holding office. Additionally, a leader’s post has often been used as a proxy for leadership in studies that have concentrated on political settings (Caprara & Zimbardo, 2004; Wolbrecht & Campbell, 2007).

A number of methodological constraints associated with the execution of cross-cultural research are also evident and likely to increase response bias, potentially affecting the reported differences found in the study.

Many models attempt to provide an outlook of what different cultures entail. Hofstede (1980), Kluckhohn and Strodtbeck (1961), Schwartz (1992) and Trompenaars (1993) have all proposed measurable value dimensions of culture. The capacity for measuring values is, however, often absent, and it is common practice to use nationality as a proxy for culture, albeit nationality does not always equate to culture. Nevertheless, in cross-cultural studies, difficulties associated with increased expenses, overseas travel, unfamiliarity with respondents and language barriers lead many researchers to engage in such practices. Indeed, the current study used nationality as a proxy for culture. Similar to other cross-cultural work, the present lack of ability to measure culture was compensated for by the availability of published value benchmarks along Hofstede’s dimensions for both Bulgaria and the UK. This advantage, and the accepted robustness of Hofstede’s model, allowed for its use and acknowledgement far beyond the academic world, in spite of its criticisms and presence of alternative options (Magala, 2009). Williamson (2002) agrees that some flaws are evident but he also notes a more plausible and satisfactory model is still absent. Of course, the recommendation for future cross-cultural research would be to incorporate a measure of culture such as the Values Survey Module (VSM-94; Hofstede, 1994) which could provide more confidence in group differences resulting from actual variations in cultural values.

Other issues related to cross-cultural research are group equivalence and measure translation. Group equivalence is difficult to accomplish due to differing cultural standards (e.g. the completion of an A-level in the UK might be substantially different from the completion of the equivalent in Bulgaria – and furthermore, such differences could reflect a blend of cultural and non-cultural effects). Subtle differences in meaning are also evident across cultures which affect the provision of valid translations. The forward and backward translation methods are considered as acceptable; however, the use of the emic approach supporting culture specific measure construction is advisable in future work.

The extent and content of socially desirable responses also vary cross-culturally (Silverthorne, 2005). The issue of impression management is not only relevant to cross-cultural research, but also to research employing politicians. In Caprara et al.’s (2003) study political leaders scored significantly higher in terms of social desirability. Such an effect was expected as political leaders are generally savvier in terms of impression management, which could cause response bias during self-assessment. Such response bias could have accounted for the style differences found here. Due to the difficulty of securing lengthy testing slots with politicians, social desirability scales were not employed in this research. Fortunately, the evident group differences with regard to some, rather than all, positive qualities suggest the lack of large social desirability effect. Nevertheless, future research should consider the use of social desirability scales, allowing researchers to control for biases deriving from dishonesty (which might be deliberate or unintended) before reporting group differences.

Additional methodological issue is that of the limited sample size. Although some of the sample sizes may seem somewhat small, they are good for the present type of research, where the targeted population is difficult to access. Moreover, the adequacy of effect sizes discussed in the results section makes the current samples and any findings related to them viable. Some might also question the sample representativeness, as for example the number of sampled female MPs from Bulgaria equated to zero. Again this is something that might need addressing in the future but as such a sample is hard to come by the analysis of associated data is still worth reporting.

### Practical Implications

Despite the shortcomings of this research a number of implications of the findings are present, such as one in the field of cross-cultural relations. The effectiveness of structures like the EU, where Bulgarian and British political leaders must work together, is often facilitated by the presence of smooth collaborations. Ensuring the latter is especially important now as we experience the turbulent Brexit Era. The current results show Bulgarian and British leaders differing in terms of the leadership styles they use. The findings reveal that the Bulgarian and British leaders were equally transformational, but the Bulgarian leaders proved more transactional and passive/avoidant, compared to the British leaders. This signifies that, in dealing with each other, British leaders must allow for Bulgarian leaders to be more task-oriented, slower to intervene and, when making decisions, more likely to be absent if needed.

Furthermore, we often group cultures in dichotomies (i.e. East, West) or separate them according to geographic, language and religious regions. Within Europe, we sometimes describe countries branded ‘the former Eastern Bloc’ as similar to each other, but different from countries west of the ‘Iron Curtain’. This often leads to result generalisation and findings of research carried out in some Eastern European cultures are applied to other cultures in the formerly referred to ‘Communist Bloc’. This may be akin to a well-known general gestalt perceptual phenomenon of ‘levelling’ within categories and ‘sharpening’ across category boundaries—often a helpful heuristic process, but a cognitive bias all the same. Bulgaria, itself, has hardly been researched, but has always been grouped with the rest of the Eastern European subset countries, which might have resulted in the formation of faulty inferences. Recently, Ardichvili and Kuchinke (2002) cited that countries such as Kazakhstan and Kyrgyzstan--both former Russian republics--are different in terms of the values they hold. Such findings propose the routine homogenous treatment of cultures with common communist experience as inappropriate. Compliance with a general categorisation could lead to misunderstandings in dealings between various leaders in multinational businesses and diverse political leaders in structures like the EU. On the other hand, acceptance of the notion of uniqueness and the need for studying cultures independently could lead to the provision of precise knowledge, aiding the development of leadership training programmes and improving EU relations. An independent study of Bulgaria, the UK or any other EU country could have implications for effectiveness, and, while many differences across Europe have not been noted, small but sometimes meaningful discrepancies could create a conflict, reduce productivity and block ‘good’ leadership.

### Theoretical Implications

The unexpected findings suggesting universality of transformational leadership behaviours amongst political leaders of differing cultures have theoretical implications. The results oppose those who described the emergence of transformational leadership as culture contingent (Jung et al., 1995; Leong & Fischer, 2011). Possible discrepancies between the current and past research outcomes could be caused by the different samples used across studies. It is possible that participant variables could moderate the relationship between transformational leadership pervasiveness and culture. For example, aspects such as leadership arena (e.g. organisational, military, religious) could provide further clarity with regard to transformational leadership universality. It could be that cross-cultural universality is limited to a particular sample of leaders. The results therefore open up additional questions and provide grounds for further in-depth exploration.

### Future Research

Future studies should endeavour to continue research into political leaders with the use of direct measures. Studying variables which might predict good leadership as well as variables potentially causing variance in the perceptions of good political leaders could inform areas such as political leader selection and political leader image management during electoral campaigns. Studying within-culture style differences in terms of additional variables such as for example political ideology and personality could further fine tune the transformational leadership theory as well as underline diversity. More work looking at the effects of mediators on the transformational leadership-culture relationship could be valuable in informing and augmenting the transformational leadership theory. Similarly, further exploration of cross-cultural differences might help explain the presence of disputes which often compromise peace.
